# Phytochemical and Safety Evaluations of Volatile Terpenoids from *Zingiber cassumunar* Roxb. on Mature Carp Peripheral Blood Mononuclear Cells and Embryonic Zebrafish

**DOI:** 10.3390/molecules25030613

**Published:** 2020-01-30

**Authors:** Raktham Mektrirat, Terdsak Yano, Siriporn Okonogi, Wasan Katip, Surachai Pikulkaew

**Affiliations:** 1 Department of Veterinary Biosciences and Public Health, Faculty of Veterinary Medicine, Chiang Mai University, Chiang Mai 50100, Thailand; raktham.m@cmu.ac.th; 2 Research Center for Pharmaceutical Nanotechnology, Chiang Mai University, Chiang Mai 50200, Thailand; okng2000@gmail.com; 3 Department of Food Animal Clinic, Faculty of Veterinary Medicine, Chiang Mai University, Chiang Mai 50100, Thailand; vetjek@gmail.com; 4 Department of Pharmaceutical Sciences, Faculty of Pharmacy, Chiang Mai University, Chiang Mai 50200, Thailand; 5 Department of Pharmaceutical Care, Faculty of Pharmacy, Chiang Mai University, Chiang Mai 50200, Thailand; wasankatip@gmail.com

**Keywords:** Zingiberaceae, essential oil, terpene, cytotoxicity, embryotoxicity, zebrafish

## Abstract

Pharmaceutical products of essential oil from *Zingiber cassumunar* Roxb. are extensively being developed, while the research on their safety is seldom documented. The aim of the present study was to evaluate the phytochemical profile and the effect of cassumunar ginger oil on cell-based assay and the zebrafish model. The essential oil was isolated from fresh rhizomes of *Z. cassumunar* using simultaneous steam-distillation. Chemical composition was analyzed using gas chromatograph coupled to a mass spectrometer (GC-MS). Effect of cassumunar ginger oil on adult carp fish peripheral blood mononuclear cells (PBMCs) was investigated using MTT assay. The embryotoxic and teratogenic effects of cassumunar ginger oil were studied in zebrafish embryos. GC-MS results showed that the essential oil was composed of sabinene (43.54%) and terpinen-4-ol (29.52%) as the major phytoconstituents. No fish PBMC cytotoxic effect was observed with the concentration less than 50 µg/mL of cassumunar ginger oil. Our results showed for the first time the embryotoxic and teratogenic effects of cassumunar ginger oil in zebrafish embryos. The result indicated that the cassumunar ginger oil induced zebrafish embryotoxicity in a concentration-dependent manner. At 500 µg/mL of cassumunar ginger oil demonstrated significantly moderated embryotoxicity within 24 h (*p* < 0.05). The survival rate of 100 µg/mL of cassumunar ginger group was markedly declined to zero at 96-h post-fertilization (log-rank test, *p* = 0.001). However, survival rates of zebrafish embryo in the 1 and 10 µg/mL cassumunar ginger groups were more than 90% throughout the trial period. Moreover, very low teratogenicity to the zebrafish embryo was also observed in 1 and 10 µg/mL of cassumunar ginger groups. Our findings suggest that there is hardly any cytotoxicity, embryotoxicity and teratogenicity at concentrations less than 10 µg/mL of cassumunar ginger oil. However, the toxicity assessment of its pharmaceutical product should prove for further consumer protection.

## 1. Introduction

The perennial angiosperm plants of family Zingiberaceae is distributed widely the tropical climatic areas including Asia, Africa and America [1]. This plant family is a significant bioresource provided various utilizations including food ingredients and spices, medicinal herbs, aromatherapy agents and textile dyes [2]. In the last decades, the phytotherapies of the plant extracts is worldwide exceeding in both human and veterinary medicines [3]. As a consequence, the rhizome of Zingiberaceous plants are a valuable source of terpenes and terpenoids, which exhibits a broad spectrum of biological effects. Plants of the Zingiber genera particularly represent useful herbal remedies under Zingiberaceous plants including *Z. cassumunar* Roxb., *Z. corallinum* Hance., *Z. nimmonii* Dalzell., *Z. officinale* Rosc., *Z. wrayi* C.K. Lim. and *Z. zerumbet* (L.) Smith. [1]. Increasingly, *Z. cassumunar* (cassumunar ginger) is commonly used as a traditional medicinal plant with treatment of a variety of illnesses in Thailand and many Asian countries [4]. The essential oils of the cassumunar ginger have previously identified the major terpenic compounds containing sabinene and terpinen-4-ol [5,6]. The modern pharmaceutical products of the functional terpenoids from cassumunar ginger have recently been developed because of its pharmacological properties including anti-inflammatory, antifungal and antibacterial efficacies [1,4,7]. The pharmacological efficacy is hindered by the low hydrophilicity of the terpene derivatives; therefore, some thermodynamically stable dosage forms are prepared for overcoming this problem [6]. Therefore, the toxicity assessments of the cassumunar ginger oils are essential to ensure the safety of phytopharmaceuticals, although the acute and the chronic oral toxicity of *Z. cassumunar* extracts using Organization of Economic Cooperation and Development (OECD) and World Health Organization (WHO) guidelines has been previously reported [8]. Geographical origin is one of the most important aspects that influence the chemical composition of plants. Unfortunately, a review of the literature revealed that there is no documented in the considering embryotoxic and teratogenic assessments of essential oil from *Z. cassumunar* from Thailand. Therefore, the present research planned to fill this gap.

In recent year, the zebrafish (*Danio rerio*) has been widely used as animal model in scientific research areas such as genetic model, cancer research and biology development [9]. The zebrafish embryo is a useful small model for investigating vertebrate development because of its rapid development, high fecundity and easy observation of transparent embryos [10]. Interestingly, the processes of development in zebrafish embryo are similar to embryogenesis of other higher vertebrates, including humans [11]. Moreover, the usage of the zebrafish model unforgettably tapers the toxicity studies using the higher vertebrate models. From these benefits, the zebrafish embryo has been considered as an alternative model for vertebrate model for toxicological assessment [9,10], as well as for new drug discovery of herb [12,13]. Recent studies on embryotoxic and developmental toxicity assessments of *Curcuma longa* Linn. extract using a zebrafish model are reported. [14]. Thus, the current investigation was used the zebrafish embryo model to assessment the embryotoxicity and teratogenicity of essential oil from *Z. cassumunar*. However, the cell-based toxicity assay should be performed for prognosticating toxicity prior to study in the whole organisms. The ability of the phytoconstituents in inhibiting the cell viability of peripheral blood mononuclear cells (PBMC) is ascertained as an indication of its systemic toxicity. Consequently, the aim of the present study was to identify chemical composition of the cassumunar ginger oil and evaluate its toxicity on in vitro adult carp PBMCs and in vivo zebrafish embryos.

## 2. Results

### 2.1. Chemical Compositions of Essential Oil

**Figure 1:**
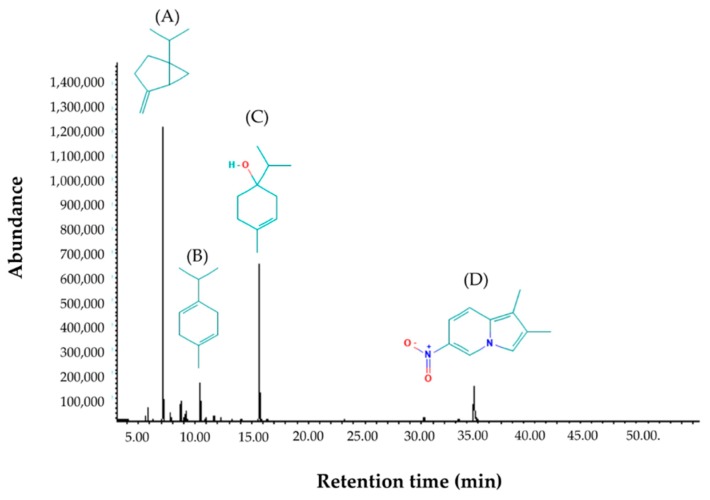
GC-MS chromatograph for essential oil isolated from *Zingiber cassumunar* Roxb. The phytoconstituents were characterized by a GC-MS method with a running time of 50 min. Chemical structures of the four major constituents identified from the cassumunar ginger oil including (**A**) Sabinene, (**B**) γ-Terpinene, (**C**) 4-Terpineol and (**D**) 1,2-Dimethyl-6-nitroindolizine.

The ginger cassumunar oil appeared as a clear pale yellowish liquid. The phytoconstituents were characterized by GC-MS method with a running time of 50 min. A list of the constituents identified in the ginger cassumunar oil and their percentage composition is shown in Table 1. The composition of essential oil was identified by terpenoids (87.97%), which consisted mainly of monocyclic monoterpenoids (87.47%) and small amounts of sesquiterpenes (0.52%). The monoterpenoids were divided into the monoterpene hydrocarbons (58.45%) and the oxygen-containing compounds (29.25%). Interestingly, an indolizidine alkaloid was present in little amounts (11.10%). The chromatogram demonstrated the presence of predominated identifiable spectra (Figure 1). The most abundant compounds were sabinene, accounting for about 43.54% of the total peak area, followed by terpinen-4-ol (29.52%), 1,2-dimethyl-6-nitroindolizine (11.10%), and γ-Terpinene (7.38%).

### 2.2. Cytotoxicity on Adult Fish PBMCS

**Figure 2:**
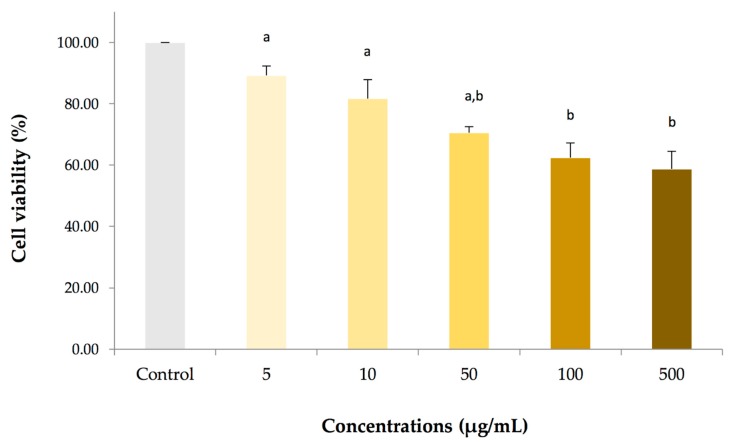
The cytotoxic effect of essential oil from *Zingiber cassumunar* Roxb. on adult carp fish PBMCs. The cells were treated with a series of concentrations in the range 5–500 µg/mL for 24 h. Percentages of cell viability were calculated from the PBMCs exposed to 0.1% DMSO (control), and 5, 10, 50, 100 and 500 µg/mL essential oils. Data represent the mean (±standard deviation, SD) of four independent experiments. Experiments were analyzed using two-way ANOVA and Tukey’s multiple comparison test. Bars not sharing a common letter are significantly different (*p* < 0.05).

To examine the essential oil from *Z. cassumunar* induced cytotoxicity, cell viability for PBMCs of adult carp fish was determined using MTT reduction assay. The cell viability was represented by the detection of enzyme mitochondrial dehydrogenase activity. The cytotoxic effect of the cassumunar ginger oil on PBMCs for 24 h was shown in Figure 2. The results summarized that the cassumunar ginger oil induced PBMCs cytotoxicity in a concentration-dependent manner. The viability rate of the PBMCs from the control group was 100%, while that of the PBMCs treated with 5, 10, 50, 100 and 500 µg/mL of the cassumunar ginger oils was 89.75 ± 3.04%, 82.32 ± 6.12%, 71.08 ± 1.92, 62.94 ± 4.73 and 59.25 ± 5.73%, respectively. The essential oil concentrations of 100 and 500 µg/mL exhibited significantly higher cytotoxicity than that of the concentration range at 5 and 10 µg/mL (*p* < 0.05). On the other hand, the cassumunar ginger oils at the concentration less than 50 µg/mL were not significantly toxic to the PBMCs by this assay.

### 2.3. Dose-response Embryotoxicity in Zebrafish

**Figure 3:**
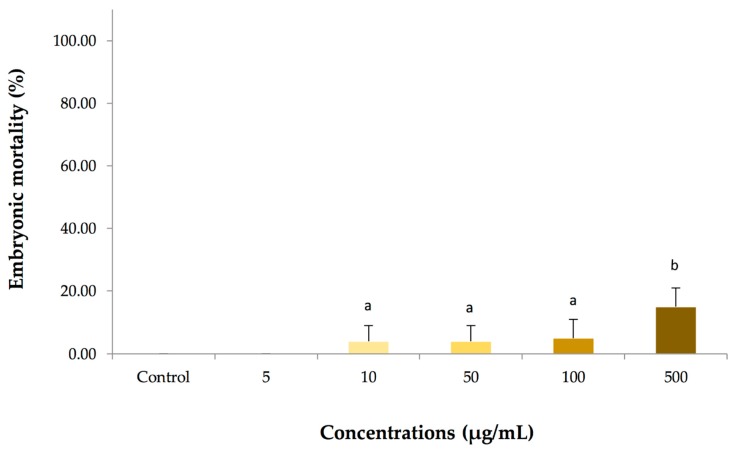
The embryotoxic effect of various concentrations of essential oil from *Zingiber cassumunar* Roxb. in embryonic zebrafish. The zebrafish embryos were treated with a series of concentrations in the range 5–500 µg/mL Percentages of embryonic mortality were calculated from zebrafish embryos exposed to 0.1% DMSO (control), and 5, 10, 50, 100 and 500 µg/mL of the cassumunar ginger oils. Data represent the mean ± SD of four independent experiments (*n* = 40 per group). Experiments were analyzed using two-way ANOVA and Tukey’s multiple comparison test. Bars not sharing a common letter are significantly different (*p* < 0.05).

The embryotoxicity was determined at multiple concentrations of the cassumunar ginger oils (5, 10, 50, 100 and 500 µg/mL) prior to evaluating its killing-kinetic effect. Absence of larvae heartbeat and coagulation of embryo were used as criteria to differentiate viable from non-viable zebrafish embryo (Figure 5B). Our results revealed that the toxic effect of cassumunar ginger oils were found to be dependent on dose. The mean mortality of zebrafish embryo at 24 h was obtained as shown in Figure 3. The results summarized that no mortality was observed in embryos exposed to 0.1% DMSO (control) and 5 µg/mL of the cassumunar ginger oils. A few mortality rates of embryos exposed treated with 10, 50, 100 µg/mL of the cassumunar ginger oils was 2.50 ± 5.00%, 2.50 ± 5.00%, and 15.00 ± 5.77%, respectively. Significantly increasing mortality rates (*p* < 0.05) were demonstrated in treatment at 500 µg/mL when compared with the low concentration of cassumunar ginger oils (≤100 µg/mL).

### 2.4. Time-kill Analysis in Zebrafish Embryos

**Figure 4:**
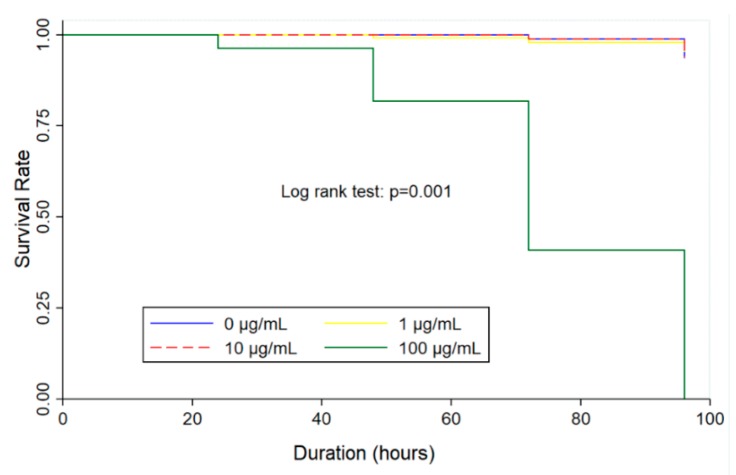
The embryotoxic effect of essential oil from *Zingiber cassumunar* Roxb. on time-killing in embryonic zebrafish. The zebrafish embryos were exposed to 0.1% DMSO (control), and 1, 10 and 100 µg/mL of the cassumunar ginger oils. Kaplan–Meier plot represents survival rate in four different groups of four independent experiments (n = 40 per group). The Log Rank test was used for statistical analysis (*p* < 0.01).

The study of dose-response embryotoxicity suggested that a value corresponding to the maximum safety concentration on zebrafish embryos are given at 100 µg/mL of the cassumunar ginger oils. Therefore, the time-kill analysis of three different concentrations of cassumunar ginger oils (1, 10 and 100 µg/mL) was performed to evaluate the kinetic killing of zebrafish embryos at 24, 48, 72 and 96 h. The Kaplan–Meier curve is used to demonstrate the survival time from a certain date to time of zebrafish embryo death (Figure 4). The result showed that the killing ability of cassumunar ginger oil was performed in a time dependent manner. However, survival rates of embryo in the control, 1 and 10 µg/mL of cassumunar ginger groups were more than 90% throughout the trial period. Whereas, the survival rate of 100 µg/mL of cassumunar ginger groups was markedly declined to zero at 96 h post-exposure observation time (log-rank test, *p* = 0.001).

### 2.5. Teratogenicity in Zebrafish

**Figure 5:**
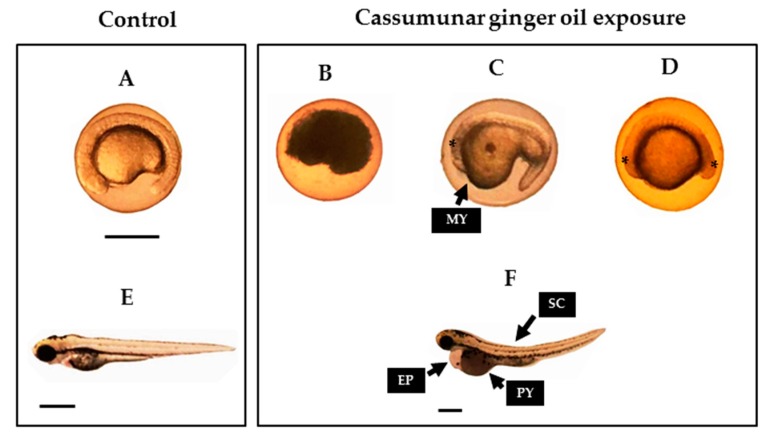
Effect of essential oil from *Zingiber cassumunar* Roxb. on typical malformations in zebrafish embryos. The zebrafish embryos were exposed to 0.1% DMSO (control), and 1, 10 and 100 of the cassumunar ginger oils. The normal morphology of zebrafish embryo and larvae exposure to filtered water and to 0.1% DMSO (control) at 24 h (**A**) and 72 h (**E**). Typical malformations caused by 100 µg/mL of cassumunar ginger oils on zebrafish embryonic development at 24 h (**B**,**C**,**D**) and 72 h (**F**). Description: coagulation (**B**) and embryo with teratogenic effect (**C**,**D**,**F**). Abbreviations: MY, malformation of yolk sac; PY, poor reabsorption of yolk sac; and SC, spinal column curving. (Scale bars = 1 µm).

To examine the essential oil from *Z. cassumunar* induced teratogenicity, the zebrafish embryos were determined at multiple concentrations of the cassumunar ginger oils (1, 10 and 100 µg/mL) during range of exposure time 24–96 h. The teratogenic effect of the cassumunar ginger oil in zebrafish embryos was shown in Table 2. The result illustrated that the malformation rate of embryonic zebrafish exposed to 10 and 100 µg/mL of cassumunar ginger oils for 24 h was 1 ± 0.8% and 24.5 ± 6.6%, respectively. The embryonic morphology was observed including the malformation of yolk sac and the malformation of head and tail development (Figure 5D). Surprisingly, accumulative abnormality to the zebrafish embryo was not found in all experiment groups at 48 h. However, some remaining embryos that were exposed to 100 µg/mL showed pericardial sac edema, the abnormality of the spinal column and the poor reabsorption of yolk sac (Figure 5F,H) with 10.5 ± 1.9% at 72 h. Mortality rate of 100% was observed at 96 h after exposure to 100 µg/mL of cassumunar ginger oils and all embryos were defined as coagulation of embryo (Figure 5B). Interestingly, no teratogenic abnormality of the zebrafish embryo was found in control and 1 µg/mL groups throughout the trial period. (Figure 5A,E).

## 3. Discussion

Terpenes and their derivatives are worldwide exceeding in both human and veterinary medicine. Fortunately, the essential oil from *Z. cassumunar* Roxb. was previously characterized as the natural terpenic compounds. The major component including sabinene (53.50%) and terpinen-4-ol (29.96%), γ-Terpinene (7.25%), and (*E*)-1-(3,4-dimethoxyphenyl) butadiene (16.16%) had found in essential oil isolated from a native Thai species of *Z. cassumunar* [5]. The phytochemical composition of essential oil from *Z. montanum* Koenig Link ex A. Dietr were also sabinene (56.34%), terpinen-4-ol (10.17%) and (*E*)-1-(3,4-dimethoxyphenyl) butadiene (14.7%) [15]. These findings are in agreement with our results on cassumunar ginger oil which the major constituents were sabinene (43.54%), terpinen-4-ol (29.52%) and γ-Terpinene (7.38%), respectively. Our results demonstrated differences in their constituents from an indolizidine alkaloid of 1,2-dimethyl-6-nitroindolizine (11.10%). On the other hand, the essential oil from *Z. cassumunar* cultivated in Malaysia showed the distinctive compounds including 6,9,9-tetramethyl-2,6,10-cycloundecatrien-1-one (60.77%) and α-caryophyllene (23.92%) [16]. The amount and phytochemicals of essential oils are decided by the plant ontogenesis; therefore, the growing season and the harvesting time are the exogenous factors affecting quality and quantity [15,17]. The extraction techniques of the distillations and the solvent extractions are also influencing both yield and quality of essential oils [18]. Moreover, the different cultivation areas affecting the phytochemical constituents of *Z. montanum* have previously been published [15].

The major component of the cassumunar ginger oil consists of monocyclic monoterpenoid with sabinene and terpinen-4-ol were well documented. Moreover, both sabinene and terpinen-4-ol exhibit a broad spectrum of biological effects [19,20]. Therefore, many efforts are development of cassumunar ginger oil as a value-added material that is useful as a pharmaceutical excipient for pharmaceutical, cosmetic and food industries [1,21]. In the most interest of the anti-inflammatory activity, attempts have recently been made to derive this cassumunar ginger oil to formulate the tropical preparations for local and systemic administrations. The topical and the transdermal deliveries of cassumunar ginger oil have been successfully developed by microemulsion, gels and patches [6,22,23,24]. Development of pharmaceutical products from cassumunar ginger oil are extensively growing, while the research on their toxicity is still limited. The acute and chronic oral toxicity of *Z. cassumunar* extract granules in Sprague–Dawley rats using OECD and WHO guidelines have been previously reported [8]. Their results demonstrated that the animals treated with the granules of *Z. cassumunar* extract were not showing the clinical signs related to toxicity in both single and chronic administration. Therefore, the oral no-observed-adverse-effect level (NOAEL) in rats was documented at 1,125 mg/kg body weight/day. Unfortunately, there is no documentation considering embryotoxic and teratogenic assessments of essential oil from *Z. cassumunar*.

The cell-based toxicity assay on adult fish PBMCs was performed for prognosticating toxicity of cassumunar ginger oil prior to study in the whole organism of zebrafish. The cytotoxicity result of the essential oil on adult fish PBMCs was in dose-dependent manner. The concentration at 5, 10 and 50 µg/mL of the essential oils showed low effect to adult fish PBMCs; whereas, the essential oil concentrations of 100 and 500 µg/mL exhibited significantly higher cytotoxicity than that of the low concentration (≤50 µg/mL). The mechanisms of cassumunar ginger oil-mediated cell death are difficult to delineate under this methodologic study. In the reaction mixture, MTT is reduced by mitochondrial enzymes of metabolically active cells and the amount of MTT formazan generated is directly proportional to the viable cell number [25]. Therefore, the MTT assay is used for measuring the screening results of cytotoxicity or proliferative effect of the medicinal substances. However, the percentage of cytotoxicity at highest concentration of the essential oils (500 µg/mL) is lower the inhibitory concentration 50% (IC_50_) in this study. Moreover, a previous study demonstrated that cytotoxicity of purified compounds from crude extract of *Z. cassumunar* has weak toxic activity with IC_50_ values of L929 cell line (1263.42 to 2857.83 µg/mL) and Vero cell line (1537.83 to 2698.45 µg/mL) [26]. These results suggest that cassumunar ginger oil has strong safety on the animal cells exposed directly.

The effects of cassumunar ginger oil on in vivo embryotoxicity or teratogenicity in zebrafish embryos were investigated. No embryonic mortality or malformation were observed in the low concentration of cassumunar ginger oil (≤10 µg/mL). At the concentration of 100 ug/mL, an increase in embryotoxicity by a time-dependent manner was observed. This corresponds to the previous finding on *Curcuma longa* Linn. extract which exhibited embryotoxicity at high concentrations (125 µg/mL) on developing zebrafish [14]. The zebrafish fed with the essential oil from *Pistacia lentiscus* Var. *chia* with high concentrations of 100 and 200 ppm also demonstrated in increased larvae mortality [27]. It is already known from many research reports that both *Z. cassumunar* and *C. longa* plants of family *Zingiberaceae* represent terpenic and phenolic structures. It can readily pass through the cytoplasmic membrane of zebrafish embryos. Effective permeability of terpenic compounds depends on its chemical structure including hydrophobicity, small size, energy of vaporization and degree of unsaturation [28]. Moreover, it is notable that outer membrane enclosing the embryo was uninterruptedly altered with advancing age of embryo development. This alteration of the protective layer has caused in opening and widening of the chorion pore channel; therefore, the increased uptake of external solutes occurred [29].

Current evidence suggests that the essential oil from *Z. cassumunar* possess certain toxic effects on embryos and development of larvae at high dosage. It should be noted that the toxicity of *Z. cassumunar* are on the basis of their isolated active phytochemical compounds. The pharmaceutical necessities use in pharmaceutical products may also cause toxicity and other disadvantage to the formulation. Therefore, the toxicity assessment of the pharmaceutical product of *Z. cassumunar* should be studied further. Among the 808 articles of the clinical efficacy and safety on *Z. cassumunar* previously was identified by a systematic review [30]. Moreover, the pharmacokinetics of the major bioactive components from *Z. cassumunar* following oral and topical administrations in rats were previously evaluated [31,32,33]. In drug development, is critical to ensure that the preclinical study properly supports the patient safety and quality of health care. Our results contribute to understanding the developmental toxicity of essential oil which has a predictive value with regard to its safety.

## 4. Materials and Methods

### 4.1. Plant Materials

The fresh rhizomes of cassumunar ginger were purchased from the local market in Chiang Mai, Thailand (Lat 18° 47′ 46.1148′′ N and Long 98° 58′ 45.3468′′ E). The voucher specimens were deposited at the Herbarium of the Faculty of Pharmacy, Chiang Mai University, Thailand. The rhizomes were cleaned with water and chopped into pieces. The sample was shade dried and powdered using a dry grinder. The powdered material was stored in light-resistant container until used in the extraction studies.

### 4.2. Distillation of Essential Oil and Chemical Characterization

The essential oil was isolated by the simultaneous steam-distillation using a Clevenger apparatus for 3 h. The essential oil obtained was dried and stored in an airtight dark bottle at −20 °C until use. Chemical characterizations of the obtained essential oil were analyzed using a gas chromatography-mass spectrometry (GC-MS) method. The GC-MS analysis was performed using a model 6890 gas chromatograph equipped with a 5973 mass-selective detector using HP-5MS column (30 m × 250 µm i.d. × 0.25 µm film thickness). The identification of each compound was based on their retention times relative to those of authentic samples and matching spectral peaks available in Wiley, NIST, and NBS mass spectral libraries. The percentage of each component was calculated based on the total area of all peaks obtained from the oil.

### 4.3. Animal and Ethic Statement

Koi carp (*Cyprinus carpio* Koi) and Zebrafish (*Danio rerio*) were obtained from the stock of the Faculty of Veterinary Medicine, Chiang Mai University. Adult fish were kept in the glass tanks using filtered tap water (pH 7.4–7.6) and held in natural light conditions. Water parameters including temperature and pH were monitored daily. The fish were fed twice daily with frozen brine shrimp in the morning and with commercial dry feed in the afternoon. Feces were siphoned out of the tanks every day. The experiment was conducted in accordance with the protocols approved by the Animal Ethics Committee, Faculty of Veterinary Medicine, Chiang Mai University (Ethic Permit No. S5/2562).

### 4.4. Fish Blood Collection and PBMC Isolation

The pooled blood samples were collected from twelve anesthetized carp fish. Each of four fish in three independent experiments were recruited into the study. About three ml of whole blood was collected by caudal venipuncture into a coagulation tubes containing a buffered sodium citrate solution. The PBMCs were isolated by Ficoll gradient centrifugation according to the manufacturer’s instructions. Briefly, a stratified sample between 6 mL of Ficoll–Hypaque and 12 mL of peripheral blood was prepared and centrifuged at 400× *g* for 20 min. the PBMCs were carefully aspirated from the Ficoll-plasma interface and washed three times with sterile phosphate-buffered saline (PBS). The cell viability was assessed by trypan blue exclusion under the light microscope.

### 4.5. In Vitro MTT Reduction Assay

Isolated PBMCs of carp fish were adjusted to 5 × 10^5^ cells/mL in RPMI 1640 supplemented with 1% heat-inactivated fetal bovine serum. The fish PBMCs were treated with various concentration of 0, 5, 10, 50, 100 and 500 µg/mL cassumunar ginger oil and incubated in 5% CO_2_ incubator under humidified conditions at 37 °C. Briefly, the solutions were freshly prepared by mixing the appropriate amount of oil and pure solvent DMSO in a sterile tube and subsequently mixing under moderate agitation using a vortex mixer until homogenous. The cytotoxicity was measured by MTT (3-(4,5-dimethylthiazol-2-yl)-2,5-diphenyl tetrazolium bromide) reduction assay at 24 h after exposure of cassumunar ginger oil. The volume of 10 µL of 5 mg/mL MTT was added into 200 µL of cell suspension and incubated for 4 h. The volume of 100 µl 0.1 M HCl in absolute isopropanol was added after incubation. The colorimetric determination of formazan product was spectrophotometrically measured at 570 nm. Cell viability was expressed as a percentage of the control culture with 0.1% DMSO.

### 4.6. Zebrafish Breeding and Embryo Acquisition

One male and two female adult zebrafishes were kept during the breeding period. Zebrafish embryos were spawned by natural mating. The fertilized eggs collected and were rinsed with sea salt egg water (60 mg/L sea salt and 2 mg/L methylene blue). Selections of fertilized egg in a stage 3–4 h postfertilization (hpf) were performed under a stereomicroscope (Nikon, Tokyo, Japan). Qualified embryos were maintained at 28.5 °C in clean petri dishes with previously egg water until analysis.

### 4.7. Zebrafish Embryonic Toxicity Test

#### 4.7.1. Dose-response Embryotoxicity

Sample embryotoxicity was determined by means of mortality rate in the zebrafish embryos. Briefly, the zebrafish embryos (*n* = 10) were transferred to individual wells of 24-well plates. The embryos were exposed to various concentrations of essential oil from *Z. cassumunar* in DMSO (100 mg/mL) to make 0, 5, 10, 50, 100 and 500 µg/mL of final concentration. The embryos were observed under a stereomicroscope at 24 h post-fertilization (hpf). The identification of death embryo was defined with coagulation of embryo or no visual heartbeat of larvae. The number of dead embryos were recorded, and the mortality rate was calculated.

#### 4.7.2. Time-killed Analysis

The killing kinetic of essential oil from *Z. cassumunar* was performed to determine the time killing rates of Zebrafish embryos. Briefly, ten fertilized eggs (*n* = 10) for each concentration of treatment essential oil from *Z. cassumunar* were studied. The experiment was performed in four independent replicates in a 24-well plate containing 2 mL of embryo media with 0.1% DMSO containing 100 μg of essential oil from *Z. cassumunar*. It was serially diluted via 10-fold serial dilution to produce 3 different concentrations of essential oil from *Z. cassumunar*. The death embryos were identified under a stereomicroscope and the time-killed analysis was studied at 24, 48, 72 and 96 h. The data was recorded, and the Kaplan–Meier curve was generated.

### 4.8. Zebrafish Teratogenicity Test

Sample teratogenicity was determined by morphological appearance and development of the zebrafish embryos. Briefly, the zebrafish embryos (*n* = 10) were transferred to individual wells of 24-well plates containing the essential oil from *Z. cassumunar* (0, 5, 10, 50, 100 and 500 µg/mL of final concentration). After incubation, the morphological defects of the exposed embryos were evaluated every 24 h under a stereomicroscope. Embryonic morphology was determined according to OECD test guidelines [34] and normal development of embryo was compared with previous described by Kimmel et al. [11]. The teratogenicity was assessed by descriptive determining the percentage of embryos or larvae with abnormality over remaining embryos.

### 4.9. Statistical Analysis

The experiments were done with four independent replications of each treatment and each killing time of study. The PBMC viability and the mortality of zebrafish embryos were compared by means of two-way analysis of variance (ANOVA) and Tukey’s multiple comparison test using R analysis program. All data are presented as mean ± standard deviation of the mean (SD), and the probabilities less than 0.05 were considered significant. Statistical analysis of killing time was performed using Stata software, version 14 (Stata-Corp, College Station, TX). Differences between the four groups in rates of time to outcome, was compared using the Kaplan–Meier curve and the log-rank test for significance.

## 5. Conclusions

This present research has indicated that the phytochemical characterization of cassumunar ginger oil has demonstrated sabinene and 4-terpineol as the major composition, along with other volatile compounds to sum a total of 11 identified phytoconstituents. Our results highlighted for the first study of embryotoxicity and teratogenicity of essential oil isolated from *Z. cassumunar* Roxb. This study provides safety information of cassumunar ginger oil in the cell-based toxicity assay and the zebrafish model. The cassumunar ginger oil at the concentration less than 50 µg/mL was not toxic to the adult fish PMMCs by MTT reduction test, whereas, no embryotoxicity and teratogenicity since 10.8% teratogenic effect is not significant. However, the toxicity assessment of its pharmaceutical product should prove for further consumer protection.

## Figures and Tables

**Table 1:** Chemical composition of essential oil from *Z. cassumunar* Roxb. obtained by GC-MS analysis. The cassumunar ginger oil was extracted from the rhizomes of *Z. cassumunar* by simultaneous steam-distillation and analyzed by GC-MS. RT: Retention time; MW: Molecular weight.

Peak	RT (min)	Component	Formula	MW (g/mol)	Amount (%)
1	5.64	α-Thujene	C_10_H_16_	136.23	0.60
2	5.83	α-Pinene	C_10_H_16_	136.23	1.58
3	7.16	Sabinene	C_10_H_16_	136.23	43.54
4	7.84	Myrcene	C_10_H_16_	136.23	1.17
5	8.74	α-Terpinene	C_10_H_16_	136.23	2.82
6	9.09	Benzene	C_6_H_6_	78.11	0.94
7	10.46	γ-Terpinene	C_10_H_16_	136.23	7.38
8	11.69	α-Terpinolene	C_10_H_16_	136.23	0.84
9	15.68	Terpinen-4-ol	C_10_H_18_O	154.25	29.52
10	30.24	*β*-Sesquiphellandrene	C_15_H_24_	204.35	0.52
11	34.63	1,2-Dimethyl-6-nitroindolizine	C_10_H_10_O_2_N_2_	190.20	11.10

**Table 2:** Teratogenic effects of essential oil from *Zingiber cassumunar* Roxb. on early development of zebrafish. The zebrafish embryos were treated to 0.1% DMSO (control), and 1, 10 and 100 µg/mL of the cassumunar ginger oils at different times of exposure (24, 48, 72 and 96 h). Descriptive data represent the mean ± SD of teratogenic embryo percentage of four independent experiments (*n* = 40 per group). NE = no surviving embryo.

Concentrations (µg/mL)	Times of Exposure (h)			
	24	48	72	96
0	0	0	0	0
1	0	0	0	0
10	1.0 ± 0.8	0	0	0
100	24.5 ± 6.6	0	10.5 ± 1.9	NE
